# Spatio-temporal analysis of childhood vaccine uptake in Nigeria: a hierarchical Bayesian Zero-inflated Poisson approach

**DOI:** 10.1186/s12887-023-04300-x

**Published:** 2023-09-29

**Authors:** Temitayo Victor Lawal, Kehinde Adebola Atoloye, Ayo Stephen Adebowale, Adeniyi Francis Fagbamigbe

**Affiliations:** 1 Department of Epidemiology and Medical Statistics, College of Medinec, University of Ibadan, Ibadan, Nigeria; 2 International Research Center of Excellence, Institute of Human Virology Nigeria, Abuja, FCT Nigeria; 3 Viable Knowledge Masters, Abuja, FCT Nigeria; 4 Viable Helpers Development Organization, Abuja, FCT Nigeria; 5 Population and Health Research Entity, Faculty of Humanities, North-West University, Mafikeng, South Africa; 6 Division of Population and Behavioural Science, School of Medicine, Health Data Science Unit, University of St Andrews, St Andrews, UK; 7 Institute of Applied Health Sciences, School of Medicine, Medicinal Sciences & Nutrition, University of Aberdeen, Aberdeen, UK; 8 Research Methods and Evaluation Unit, Institute for Health & Wellbeing, Coventry University, Coventry, UK

**Keywords:** Child vaccination, Immunization, Bayesian, Multilevel, MCMC, Zero-inflated Poisson, Nigeria

## Abstract

**Background:**

Globally, child mortality and morbidity remain a serious health challenge and infectious diseases are the leading causes. The use of count models together with spatial analysis of the number of doses of childhood vaccines taken is limited in the literature. We used a Bayesian zero-inflated Poisson regression model with spatio-temporal components to assess the number of doses of childhood vaccines taken among children aged 12–23 months and their associated factors.

**Methods:**

Data of 19,564 children from 2003, 2008, 2013 and 2018 population-based cross-sectional Nigeria Demographic and Health Survey were used. The childhood vaccines include one dose of Bacillus-Calmette-Guérin; three doses of Diphtheria-Pertussis-Tetanus; three doses of Polio and one dose of Measles. Uptake of all nine vaccines was regarded as full vaccination. We examined the multilevel factors associated with the number of doses of childhood vaccines taken using descriptive, bivariable and multivariable Bayesian models. Analysis was conducted in Stata version 16 and R statistical packages, and visualization in ArcGIS.

**Results:**

The prevalence of full vaccination was 6.5% in 2003, 14.8% in 2008, 21.8% in 2013 and 23.3% in 2018. Full vaccination coverage ranged from 1.7% in Sokoto to 51.9% in Anambra. Factors associated with the number of doses of childhood vaccines taken include maternal age (adjusted Incidence “risk” Ratio (aIRR) = 1.05; 95% Credible Interval (CrI) = 1.03–1.07) for 25–34 years and (aIRR = 1.07; 95% CrI = 1.05–1.10) for 35–49 years and education: (aIRR = 1.11, 95% CrI = 1.09–1.14) for primary and (aIRR = 1.16; 95% CrI = 1.13–1.19) for secondary/tertiary education. Other significant factors are wealth status, antenatal care attendance, working status, use of skilled birth attendants, religion, mother’s desire for the child, community poverty rate, community illiteracy, and community unemployment.

**Conclusion:**

Although full vaccination has remained low, there have been improvements over the years with wide disparities across the states. Improving the uptake of vaccines by educating women on the benefits of hospital delivery and vaccines through radio jingles and posters should be embraced, and state-specific efforts should be made to address inequality in access to routine vaccination in Nigeria.

**Supplementary Information:**

The online version contains supplementary material available at 10.1186/s12887-023-04300-x.

## Introduction

One of the most cost-effective public health interventions and major success stories of global health in achieving a reduction of child morbidity and mortality is vaccination. Vaccination is a simple, safe and efficient way of protecting individuals from harmful diseases and a very vital tool of primary health care [1]. Vaccination saves millions of lives yearly and reduces the overall “risk” of getting a disease by building protection in the body’s natural immune system, thereby helping people of all ages to live longer and healthier lives [1, 2]. The ultimate aim of vaccination programs is to achieve a reduction in the incidence of vaccine-preventable diseases by ensuring a high level of coverage and vaccine administration at appropriate ages and recommended intervals [3].

Globally, child morbidity and mortality are among the most serious health challenges, while infectious diseases are leading causes of under-five deaths [4, 5]. In 2021, the child mortality rate worldwide was 39 per 1,000 live birth, the highest of whom were residents in sub-Saharan African countries like Somalia, Niger, Nigeria, and Chad with 117, 115, 111, and 107 per 1,000 live birth, respectively [5, 6]. Low-income, lower- and upper-middle-income countries were reported to have an average of 68, 44 and 12 per 1,000 live birth, sub-Saharan African countries have about 73 per 1,000 live birth and the least developed countries were reported to have 62 deaths per 1,000 live births [5, 6].

Although factors associated with childhood deaths vary between countries, reducing child morbidity and mortality rates remains an urgent concern, as it is a major indicator for assessing a nation’s progress and development [7, 8]. Researchers have established an association between vaccine uptake and child health outcomes [9–14]. However, such studies modelled childhood vaccination as a binary outcome and the observed geographical inequality in coverage based on demographic factors.

This approach does not account for the number of vaccines taken, because the use of binary logistics regression relies on dichotomous outcomes, where vaccine uptake was in two categories: either “none” versus “at least one” or “all” versus “not all”. This approach undercounts the number of doses of childhood vaccines taken. Rather, count models will be more appropriate in the identification of factors associated with the number of doses of childhood vaccines taken. Moreover, failure to account for the hierarchical nature of the associated factors as well as the spatial and temporal distribution of the number of doses of childhood vaccines taken may undermine the reliability of the findings. 

In Nigeria, researchers have also explored factors associated with vaccination coverage [12, 14–17]. Demographic and socio-economic factors such as child-specific, parental and household characteristics have been identified as important predictors of child vaccination uptake [10, 12, 18, 19]. Birth order, age, and place of birth of a child are other factors that influence vaccination uptake [19–21]. Moreover,  parental factors such as literacy, media exposure and educational attainment have also been found to have a strong relationship with vaccination uptake [22].

We hypothesized that the number of doses of childhood vaccines taken varies across individual-, community- and state-level factors. The knowledge of factors associated with the number of childhood vaccination received and the spatial and temporal distribution of the level of the uptakes are very useful in providing evidence-based information for child and maternal health programmers. Therefore, this study aimed to explore the Spatio-temporal distribution and the factors associated with the number of doses of childhood vaccine uptake among children aged 12–23 months from 2003 to 2018 in Nigeria.

## Materials and methods

### Study area

The study area is Nigeria, the most populous country in Africa. In Nigeria, the National Primary Health Care Development Agency (NPHCDA) is responsible for controlling vaccine-preventable diseases through the provision of vaccines and its rules and guidelines. The National Social Mobilization Working Group is nationally responsible for the development of communication strategies and interventions, with State and Local Government representation. These groups carry out campaigns, alongside other private and public health organizations regularly, at intervals or as the need arises.

### Study population

The study population of participants consists of children aged 12–23 months, which represents the youngest cohort of children who are supposed to take all vaccines recommended in the first year of life.

### Study design

The study used data from a cross-sectional and nationally representative household sample survey for Nigeria; pooled from 4 consecutive datasets of the National Demographic and Health Survey (NDHS) (a population cross-sectional survey) conducted in the years 2003, 2008, 2013 and 2018.

### Sampling

The NDHS used a multi-stage, stratified sampling design for data collection with the clusters as the primary sampling units and households as sampling units. In the first stage, clusters (enumeration areas) were selected from already identified rural and urban local government areas. The second stage involved the selection of households within the selected clusters using the latest sampling frame constructed by the National Population Commission which had been used for Nigeria Population Census. All eligible women of reproductive age (15–49 years) living in the selected households were interviewed. Sampling weights were added to account for the unequal probability of selection at the cluster levels and non-response, since the samples were not self-weighted . These weights helped to minimize non-response and selection biases.

### Study variables

#### Dependent variable

The data on the uptake of all the 9 doses of the 4 vaccines were processed and merged into one variable to give the number of doses of childhood vaccines a child took. The doses are; one dose of Bacillus-Calmette-Guérin (BCG) vaccine, three doses of Diphtheria-Pertussis-Tetanus (DPT) vaccine, three doses of Polio (including Oral Polio Vaccine at birth) vaccine, and one dose of Measles vaccine. Our dependent variable is, therefore, the number of doses of childhood vaccines taken by the children.

#### Independent variables

Explanatory variables were selected based on findings in the literature [10, 12, 19, 23] and the availability of data at individual, community and state levels. Three levels of explanatory variables were used for the hierarchical nature of the study:

Individual variables such as indicators of socio-demographic characteristics of the child and mother including mother’s age, educational level, religion, working status, desire for the last child (wanted or not), skilled birth attendant presence on delivery, exposure to media, birth order, wealth status (divided into three quartiles), number of antenatal care attendance, sex of the child, and healthcare decision-maker, were included in the analysis. Exposure to media, in this study, was defined as the mother’s access to information through any newspapers/magazines, radio or television (i.e., if the mother reads or watches any, at least once a week).

Community-level variables (place of residence, community poverty rate, illiteracy rate and unemployment) and State level variables (rural population and health facilities per 10,000 population) were included in the model.

### Data management

Data were extracted from the DHS website and pooled for analysis. Before the multivariate analysis, children with missing information on vaccination uptake were removed (a total of 1,698 samples were dropped from the dataset). Mothers were asked to either show the vaccination cards of their children or verbally report if they had taken the vaccines. However, respondents who reported “don’t know” and “no vaccination” were categorized as not having received the vaccination for their children. A weighted univariate and bivariate (Chi-square) analysis was done in Stata version 16 to adjust for unequal cluster sizes, and stratifications and ensure that the findings are representative of the population of interest for each state [2]. Statistical significance was set to 0.05. ArcGIS PRO version 2.8 was used to draw maps, while BayesX in R was used for Spatiotemporal regression and to create the posterior maps.

### Statistical analysis

Weighted descriptive and univariate analyses were carried out in the study. To identify the association/relationship between the individual-, community- (contextual) and state-level (compositional) characteristics and vaccination, a chi-square test was carried out on the association between explanatory variables and the number of doses of childhood vaccines taken using the Stata version 16; after which the count modelling was carried out in BayesX package of R.

Before the regression modelling, a multicollinearity test was performed on the variables and a variable with a high correlation (variance inflation factor > 10) was dropped from the multivariable analysis. Specifically, the variable named “total number of children” was correlated with the “birth order of the child”. We, therefore, dropped the “total number of children” from the analysis. Using all the 3-level model for the count response, which was defined as children, i, who took vaccination (at level 1), from a community, j (at level 2) and living in a state, k (at level 3), the models the study worked with is the generalized linear mixed model (GLMM) with both fixed and random effects.

Two count modelling strategies were considered, including the Bayesian Poisson and Zero-inflated Poisson. Previous studies have compared the performance of the frequentist and Bayesian and found the Bayesian approach to be more robust and gave a better result for convergence assessment [24–29]. We treated the doses of different vaccines as they were taken at different times, which could bring about differences in their uptakes as they are not forced to take consecutive doses haven taken the first doses of each vaccine.

In this study, our outcomes are counting outcomes (number of doses) for each child because the number of doses received by children differs. Count models using the frequentist approach have been used to model the number of children ever born and other fertility outcomes [30, 31], HIV care [32] and the number of school suspensions [33].

Model selection and comparison were done using the − 2 Log-Likelihood, Watanambe Information Criterion (WAIC) and Leave-one-out Information Criterion (LOOIC) – an additional file shows this in more detail [see Additional file 1]). The Zero-inflated Poisson model performed better judging with the lowest value of the measure of goodness.

The fully adjusted model was then used to control for the effects of all the levels (individual-, community-, and state-level characteristics). The median posterior estimates of the models and standard error were presented. Model selection was based on the model’s goodness of fit parameters stated earlier.

In this study, the presence of non-linear effects for some covariates indicates that strictly linear predictors cannot be assumed. The geographical patterns of vaccine uptake and the possible non-linear effects were simultaneously explored using a hierarchical model that controlled for spatial dependence and nonlinear time-varying effects of covariates. The simple model is of the form:

$${{\eta }}_{{ijk}}={\beta }_{0}+ \sum _{p=1}^{P}{\beta }_{p}{x}_{pijk}+{U}_{0jk}+ {V}_{0k}$$

Where η is the link function; ${\beta }_{0}$ is the intercept; ${\beta }_{p}$ is the regression coefficient for the p parameters; ${x}_{pijk}$ are the covariates; ${U}_{0jk}$ is the random component for children from community j, in state k; ${V}_{0k}$ is the random component for children in state k. The linear predictor is a flexible log link function with temporal and spatial effects:

$${{\eta }}_{{ijk}}={f}_{1}{x}_{i1}+{f}_{spat}\left({s}_{i}\right)+ \sum _{p=1}^{P}{\beta }_{p}{x}_{pijk}+{U}_{0jk}+ {V}_{0k}$$

Where ${f}_{1}$ is the non-linear smoothed effect of the metrical covariate of mothers age, and ${f}_{spat}\left({s}_{i}\right)$ is the effect of the spatial covariate labelling the state in Nigeria.

## Results

Table 1 presents the distribution of participants by their socio-demographic characteristics. The variables were distributed into individual-, community-, and state-level characteristics, The model employed for the number of doses of childhood vaccines taken in this study is the Bayesian Zero-Inflated Poisson three-level variance components model.

**Table 1:** Weighted Distribution of respondent’s characteristics by survey year

	2003 (n = 1151) [n (%)]	2008 (n = 5570) [n (%)]	2013 (n = 6281) [n (%)]	2018 (n = 6562) [n (%)]	Total (n = 19,564) [n (%)]
**Level 1 (Individual Level Characteristics)**					
**Age (Median; (IQR))years**	**(28; (23–33))**				
15–24 years	345 (30.0)	1,618 (29.1)	1,795 (28.6)	1,877 (28.6)	5,635 (28.8)
25–34 years	555 (48.2)	2,720 (48.8)	3,063 (48.8)	3,207 (48.9)	9,545 (48.8)
35–49 years	251 (21.8)	1,232 (22.1)	1,423 (22.7)	1,478 (22.5)	4,384 (22.4)
**Level of Education**					
No formal education	550 (47.8)	2,779 (49.9)	2,862 (45.6)	2,872 (43.8)	9,063 (46.3)
Primary education	297 (25.8)	1,257 (22.6)	1,231 (19.6)	983 (15.0)	3,768 (19.3)
Secondary education	265 (23.0)	1,261 (22.6)	1,736 (27.6)	2,175 (33.5)	5,437 (28.0)
Tertiary education	39 (3.4)	273 (4.9)	452 (7.2)	532 (8.1)	1,296 (7.2)
**Religion**					
Christian	231 (20.6)	2,407 (44.2)	2,403 (37.9)	2,503 (37.5)	7,544 (38.5)
Islam	116 (14.9)	2,960 (54.4)	3,843 (60.6)	4,146 (62.0)	11,116 (56.7)
Others	724 (64.5)	79 (1.5)	101 (1.6)	34 (1.0)	938 (4.8)
**Working Status**					
Not currently working	427 (37.1)	2,040 (36.6)	1,931 (30.7)	2,201 (33.5)	6,599 (33.7)
Currently Working	724 (62.9)	3,530 (63.4)	4,350 (69.3)	4,361 (66.5)	12,965 (66.3)
**Wanted child**					
Wanted then	967 (84.0)	4,960 (89.1)	5,550 (88.4)	5,648 (86.1)	17,125 (87.5)
Did not want then	184 (16.0)	610 (11.0)	731 (11.6)	914 (13.9)	2,439 (12.5)
**Attendant on delivery**					
Unskilled	714 (62.0)	3,851 (69.1)	3,885 (61.9)	3,823 (58.3)	12,273 (62.7)
Skilled	437 (38.0)	1,719 (30.9)	2,396 (38.2)	2,739 (41.7)	7,291 (37.3)
**Exposure to media**					
Not exposed at all	290 (25.2)	2,000 (35.9)	2,218 (35.3)	2,628 (40.1)	7,136 (36.5)
Partially exposed	530 (46.1)	2,078 (37.3)	2,600 (41.4)	2,740 (41.8)	7,948 (40.6)
Very Exposed	331 (28.8)	1,492 (26.8)	1,463 (23.3)	1,194 (18.2)	4,480 (22.9)
**Birth order of child**	**(3; (2–5))**				
First	212 (208)	1,033 (18.6)	1,223 (19.5)	1,250 (19.1)	3,718 (19.0)
Second	208 (18.1)	937 (16.8)	1,075 (17.1)	1,209 (18.4)	3,429 (17.5)
Third	166 (14.4)	832 (14.9)	893 (14.2)	1,022 (15.6)	2,913 (14.9)
Fourth and above	565 (49.1)	2,768 (49.7)	3,090 (49.2)	3,081 (47.0)	9,504 (48.6)
**Wealth Status**					
Bottom 33%	306 (26.6)	1,936 (34.8)	2,169 (34.5)	2,111 (32.2)	6,522 (33.3)
Average	484 (42.1)	1,908 (34.3)	2,023 (32.2)	2,106 (32.1)	6,521 (33.3)
Top 33%	361 (31.4)	1,726 (31.0)	2,089 (33.3)	2,345 (35.7)	6,521 (33.3)
**Number of ANC visit**	**(4; (0–7))**				
No visit	460 (40.0)	2,843 (51.0)	2,508 (39.9)	1,974 (30.1)	7,785 (39.8)
1–3 visits	158 (13.7)	584 (10.4)	707 (11.3)	1,048 (16.0)	2,497 (12.8)
4–7 visits	258 (22.4)	1,157 (20.8)	1,578 (25.1)	2,405 (36.7)	5,398 (27.6)
8 or more visits	275 (23.9)	986 (17.7)	1,488 (23.7)	1,135 (17.3)	3,884 (19.9)
**Sex of the child**					
Male	586 (50.9)	2,792 (50.1)	3,302 (52.6)	3,411 (52.0)	10.091 (51.6)
Female	565 (49.1)	2,778 (49.9)	2,979 (47.4)	3,151 (48.0)	9,473 (48.4)
**Healthcare decision maker**					
Self	144 (12.5)	395 (7.1)	313 (5.0)	511 (7.8)	1,363 (7.0)
Husband alone	831 (72.2)	3,177 (57.0)	3,731 (59.4)	3,753 (57.2)	11,492 (58.7)
Joint	117 (10.2)	1,723 (30.9)	1,879 (29.9)	1,920 (29.3)	5,639 (28.8)
Other/Unknown	59 (5.1)	275 (4.9)	358 (5.7)	378 (5.8)	1,070 (5.5)
**Level 2 (Community Level Characteristics)**					
**Place of residence**					
Urban	430 (37.4)	1,478 (26.6)	2,064 (32.9)	2,259 (34.4)	6,231 (31.9)
Rural	721 (62.6)	4,092 (73.5)	4,217 (67.1)	4,303 (65.6)	13,333 (68.2)
**Community poverty rate**					
Low	225 (19.6)	1634 (29.3)	1795 (28.6)	2967 (45.2)	6621 (33.8)
Average	518 (45.0)	2038 (36.6)	2156 (34.3)	1737 (26.5)	6449 (33.0)
High	408 (35.5)	1898 (34.1)	2330 (37.1)	1858 (28.3)	6494 (33.2)
**Community illiteracy rate**					
Low	116 (10.1)	1581 (28.4)	1843 (29.3)	2986 (45.5)	6526 (33.4)
Average	587 (51.0)	2073 (37.2)	2009 (32.0)	1862 (28.4)	6531 (33.4)
High	448 (38.9)	1916 (34.4)	2429 (38.7)	1714 (26.1)	6507 (33.3)
**Community unemployment**					
Low	98 (8.5)	1396 (25.1)	1832 (29.2)	3362 (51.2)	6688 (34.2)
Average	340 (29.5)	1951 (35)	2248 (35.8)	1846 (28.1)	6385 (32.6)
High	713 (62)	2223 (39.9)	2201 (35)	1354 (20.6)	6491 (33.2)
**Level 3 (State Level Characteristics)**					
**Rural proportion**					
Low rural proportion	191 (16.6)	1008 (18.1)	1166 (18.6)	1262 (19.2)	3627 (18.5)
Average rural proportion	351 (30.5)	1403 (25.2)	1466 (23.3)	1623 (24.7)	4843 (24.8)
High rural proportion	609 (52.9)	3159 (56.7)	3649 (58.1)	3677 (56.0)	11,094 (56.7)
**Health facility per 100,000**					
< 15	271 (23.5)	1327 (23.8)	1567 (25.0)	1625 (24.8)	4790 (24.5)
15–25	683 (59.3)	3158 (56.7)	3522 (56.1)	3576 (54.5)	10,939 (55.9)
> 25	197 (17.1)	1085 (19.5)	1192 (19.0)	1361 (20.7)	3835 (19.6)
**Distal Characteristic**					
**Region of residence**					
North Central	201 (17.5)	938 (16.8)	922 (14.7)	1135 (17.3)	3196 (16.3)
North East	268 (23.3)	1271 (22.8)	1277 (20.3)	1433 (21.8)	4249 (21.7)
North West	357 (31.0)	1583 (28.4)	1932 (30.8)	1889 (28.8)	5761 (29.5)
South East	99 (8.6)	497 (8.9)	605 (9.6)	739 (11.3)	1940 (9.9)
South South	111 (9.6)	641 (11.5)	788 (12.6)	667 (10.2)	2207 (11.3)
South West	115 (10.0)	640 (11.5)	757 (12.1)	699 (10.7)	2211 (11.3)

About half (49%) of the mothers were aged 25–34 years. Only 7% of the respondents had tertiary education, while a larger percentage (46%) of the women had no formal education; this pattern was also observed across each of the interview years. The percentage of women who had a skilled birth attendant present at delivery was 37%, and about 36% were not exposed to media at all (Table 1).

**Fig. 1:**
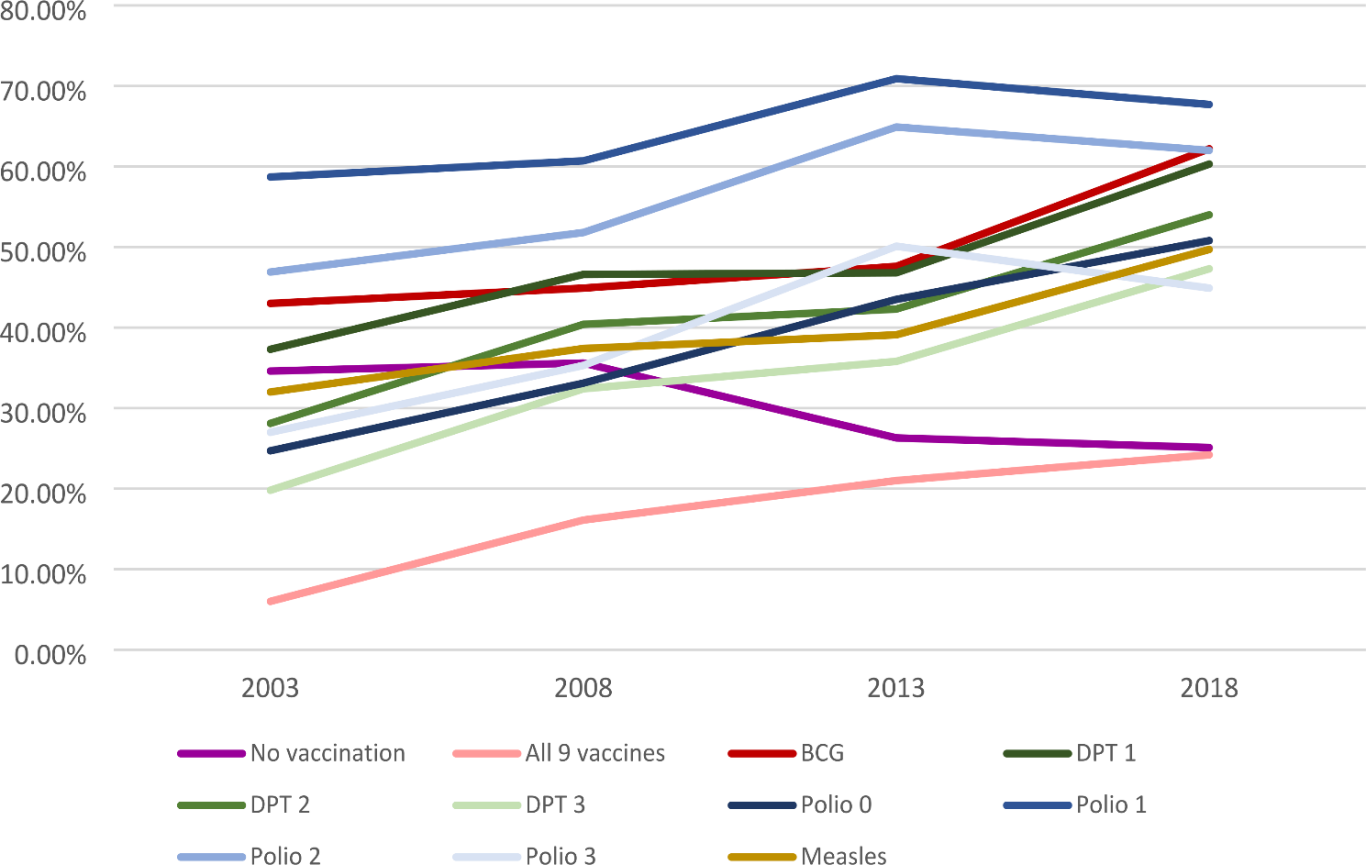
Trend of number of doses of the vaccines among children between 2003 and 2018

**Fig. 2:**
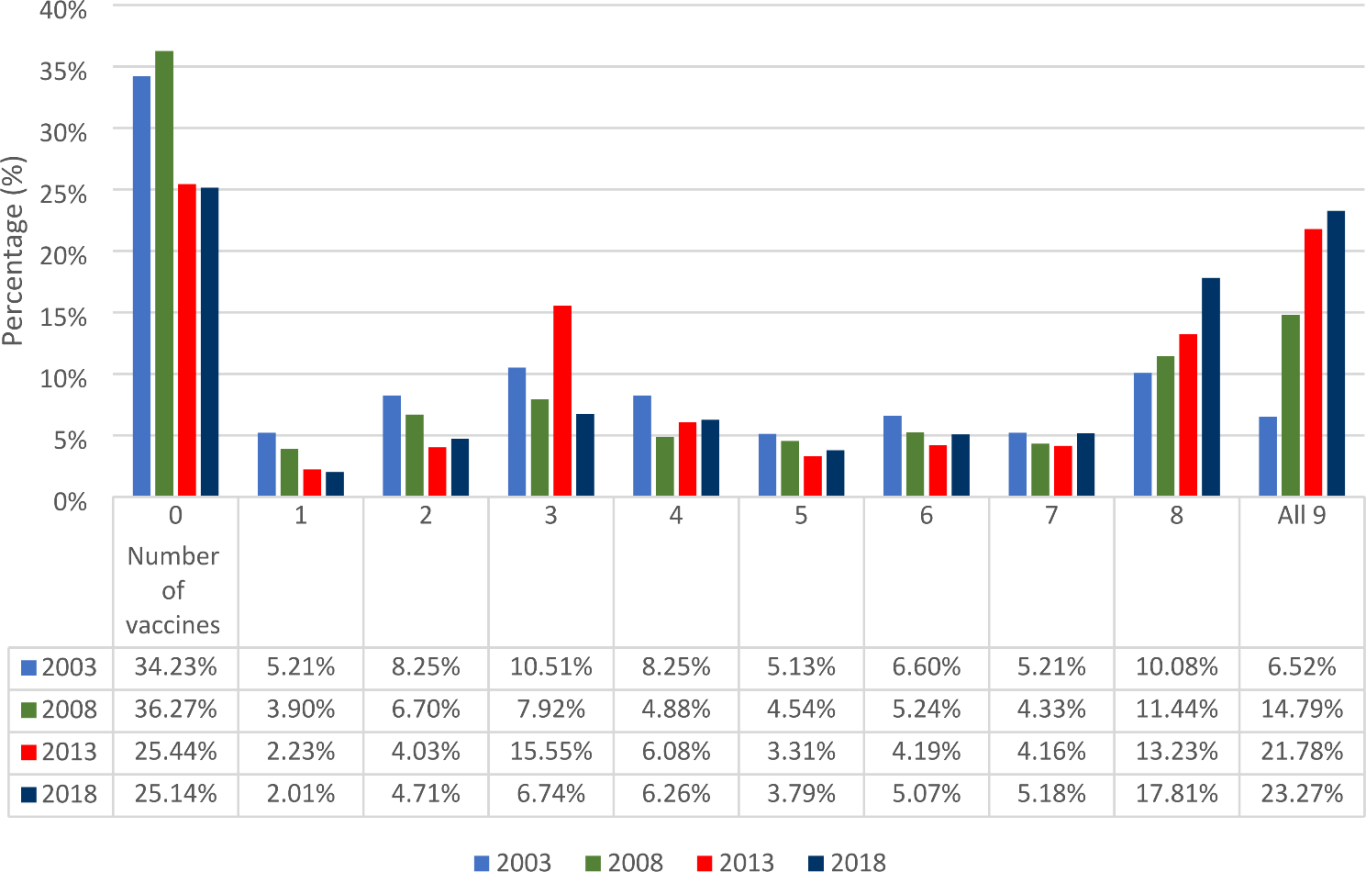
Percentage distribution of number of doses of the vaccines taken between 2003 and 2018

Prevalence of full vaccination was 6.5% in 2003, 14.8% in 2008, 21.8% in 2013 and 23.3% in 2018 (Table 2). The percentage of children who took zero number of vaccines was high among women with no ANC visit (49.1%), women with no formal education (42.7%), women of other religion (41.3%), women in the bottom 33% of wealth status (40.9%), and women not exposed to mass media at all (40.0%) as shown in Table 2; Fig. 1. Figure 1 shows the percentage of children who took each of the vaccines across the survey years. The percentage of children that took no vaccine across each of the years dropped from 35% to 2003 to 25% in 2018. Figure 2 presents the number of doses of childhood vaccines received among all the cohorts of children in the study. The distribution of vaccine uptake was similar across the survey years.

**Table 2:** Weighted distribution of respondent’s characteristics and number of doses of childhood vaccines taken

	Number of Childhood Vaccines Taken [n(%)]										${\varvec{\chi }}^{2}$ p-value
	No dose	One	Two	Three	Four	Five	Six	Seven	Eight	All Nine	
**Level 1 (Individual Level Characteristics)**											
**Age**											
15–24 years	1863 (33.1)	193 (3.4)	350 (6.2)	649 (11.5)	344 (6.1)	239 (4.2)	247 (4.4)	280 (5.0)	678 (12.0)	792 (14.1)	< 0.001
25–34 years	2519 (26.4)	244 (2.6)	469 (4.9)	877 (9.2)	548 (5.7)	356 (3.7)	508 (5.3)	421 (4.4)	1465 (15.4)	2138 (22.4)	
35–49 years	1280 (29.2)	112 (2.6)	211 (4.8)	455 (10.4)	268 (6.1)	174 (4.0)	209 (4.8)	201 (4.6)	610 (13.9)	864 (19.7)	
**Level of Education**											
No formal education	3865 (42.7)	395 (4.4)	677 (7.5)	1477 (16.3)	642 (7.1)	315 (3.5)	332 (3.7)	272 (3.0)	526 (5.8)	562 (6.2)	< 0.001
Primary education	954 (25.3)	92 (2.4)	207 (5.5)	308 (8.2)	231 (6.1)	203 (5.4)	248 (6.6)	229 (6.1)	593 (15.7)	703 (18.7)	
Secondary education	745 (13.7)	59 (1.1)	134 (2.5)	185 (3.4)	258 (4.8)	224 (4.1)	324 (6.0)	346 (6.4)	1329 (24.4)	745 (13.7)	
Tertiary education	98 (7.6)	3 (0.2)	12 (0.9)	11 (0.9)	29 (2.2)	27 (2.1)	60 (4.6)	55 (4.2)	305 (23.5)	98 (7.6)	
**Religion**											
Christian	1354 (17.3)	109 (1.4)	223 (2.8)	286 (3.7)	332 (4.2)	303 (3.9)	471 (6.0)	485 (6.2)	1767 (22.5)	2512 (32.0)	< 0.001
Islam	3901 (36.3)	378 (3.5)	700 (6.5)	1588 (14.8)	750 (7.0)	428 (4.0)	450 (4.2)	393 (3.7)	915 (8.5)	1234 (11.5)	
Others	407 (41.3)	62 (6.3)	107 (10.9)	107 (10.9)	78 (7.9)	38 (3.9)	43 (4.4)	24 (2.4)	71 (7.2)	48 (4.9)	
**Working Status**											
Not currently working	2522 (38.2)	194 (2.9)	381 (5.8)	747 (11.3)	388 (5.9)	212 (3.2)	264 (4.0)	253 (3.8)	675 (10.2)	963 (14.6)	< 0.001
Currently Working	3140 (24.2)	355 (2.7)	649 (5.0)	1234 (9.5)	772 (6.0)	557 (4.3)	700 (5.4)	649 (5.0)	2078 (16.0)	2831 (21.8)	
**Wanted child**											
Wanted then	5131 (30.0)	496 (2.9)	922 (5.4)	1836 (10.7)	1039 (6.1)	654 (3.8)	813 (4.8)	758 (4.4)	2301 (13.4)	3175 (18.5)	< 0.001
Did not want child then	531 (21.8)	53 (2.2)	108 (4.4)	145 (5.9)	121 (5.0)	115 (4.7)	151 (6.2)	144 (5.9)	452 (18.5)	619 (25.4)	
**Attendant on delivery**											
Unskilled	4631 (37.8)	474 (3.9)	862 (7.0)	1721 (14.1)	844 (6.9)	481 (3.9)	520 (4.3)	461 (3.8)	1082 (8.8)	1169 (9.6)	< 0.001
Skilled	1011 (13.9)	75 (1.0)	166 (2.3)	257 (3.5)	316 (4.3)	287 (3.9)	444 (6.1)	441 (6.1)	1669 (22.9)	2625 (36.0)	
**Exposure to media**											
Not exposed at all	2851 (40.0)	258 (3.6)	516 (7.2)	999 (14.0)	472 (6.6)	267 (3.7)	291 (4.1)	253 (3.6)	603 (8.5)	626 (8.8)	< 0.001
Partially exposed	2153 (27.1)	238 (3.0)	391 (4.9)	807 (10.2)	494 (6.2)	338 (4.3)	408 (5.1)	381 (4.8)	1135 (14.3)	1603 (20.2)	
Very Exposed	658 (14.7)	53 (1.2)	123 (2.8)	175 (3.9)	194 (4.3)	164 (3.7)	265 (5.9)	268 (6.0)	1015 (22.7)	1565 (34.9)	
**Birth order of child**											
First	971 (26.1)	82 (2.2)	172 (4.6)	272 (7.3)	194 (5.2)	137 (3.7)	186 (5.0)	183 (4.9)	597 (16.1)	924 (24.9)	< 0.001
Second	919 (26.8)	86 (2.5)	160 (4.7)	296 (8.6)	186 (5.4)	129 (3.8)	160 (4.7)	158 (4.6)	554 (16.2)	781 (22.8)	
Third	755 (25.9)	71 (2.4)	151 (5.2)	291 (10.0)	168 (5.8)	124 (4.3)	146 (5.0)	157 (5.4)	445 (15.3)	605 (20.8)	
Fourth and above	3017 (31.7)	310 (3.3)	547 (5.8)	1122 (11.8)	612 (6.4)	379 (4.0)	472 (5.0)	404 (4.3)	1157 (12.2)	1484 (15.6)	
**Wealth Status**											
Bottom 33%	33 (40.9)	2666 (4.6)	297 (8.0)	520 (16.7)	1086 (7.0)	455 (3.5)	225 (3.8)	245 (3.0)	196 (6.9)	448 (5.9)	< 0.001
Average	2022 (31.0)	169 (2.6)	350 (5.4)	670 (10.3)	419 (6.4)	313 (4.8)	352 (5.4)	349 (5.4)	890 (13.7)	987 (15.1)	
Top 33%	33 (14.9)	974 (1.3)	83 (2.5)	160 (3.5)	225 (4.4)	286 (3.5)	231 (5.6)	367 (5.5)	357 (21.7)	1415 (37.2)	
**Number of ANC visits**											
No visit	3522 (49.1)	307 (4.3)	537 (7.5)	1115 (15.5)	418 (5.8)	194 (2.7)	196 (2.7)	149 (2.1)	372 (5.2)	370 (5.2)	< 0.001
1–3 visits	627 (25.1)	82 (3.3)	175 (7)	305 (12.2)	185 (7.4)	142 (5.7)	138 (5.5)	138 (5.5)	330 (13.2)	375 (15.0)	
4–7 visits	919 (17.0)	105 (2.0)	221 (4.1)	394 (7.3)	364 (6.7)	261 (4.8)	334 (6.2)	362 (6.7)	994 (18.4)	1444 (26.8)	
8 or more visits	594 (13.2)	55 (1.2)	97 (2.2)	167 (3.7)	193 (4.3)	172 (3.8)	296 (6.6)	253 (5.6)	1057 (23.6)	1605 (35.8)	
**Sex of the child**											
Male	2990 (29.6)	271 (2.7)	503 (5.0)	1000 (9.9)	581 (5.8)	384 (3.8)	498 (4.9)	484 (4.8)	1417 (14.0)	1963 (19.5)	0.210
Female	2672 (28.2)	278 (2.9)	527 (5.6)	981 (10.4)	579 (6.1)	385 (4.1)	466 (4.9)	418 (4.4)	1336 (14.1)	1831 (19.3)	
**Healthcare decision maker**											
Self	278 (20.4)	27 (2.0)	60 (4.4)	68 (5.0)	82 (6.0)	66 (4.8)	75 (5.5)	80 (5.9)	270 (19.8)	357 (26.2)	< 0.001
Husband alone	4012 (34.9)	392 (3.4)	720 (6.3)	1501 (13.1)	744 (6.5)	427 (3.7)	509 (4.4)	435 (3.8)	1210 (10.5)	1542 (13.4)	
Joint	1112 (19.7)	98 (1.7)	212 (3.8)	329 (5.8)	279 (5.0)	226 (4.0)	311 (5.5)	331 (5.9)	1099 (19.5)	1642 (29.1)	
Other/Unknown	260 (24.3)	32 (3.0)	38 (3.6)	83 (7.8)	55 (5.1)	50 (4.7)	69 (6.5)	56 (5.2)	174 (16.3)	253 (23.6)	
**Level 2 (Community Level Characteristics)**											
**Place of residence**											
Urban	1222 (19.6)	109 (1.8)	178 (2.9)	295 (4.7)	291 (4.7)	235 (3.8)	325 (5.2)	314 (5.0)	1189 (19.1)	2073 (33.3)	< 0.001
Rural	4440 (33.3)	440 (3.3)	852 (6.4)	1686 (12.7)	869 (6.5)	534 (4.0)	639 (4.8)	588 (4.4)	1564 (11.7)	1721 (12.9)	
**Community poverty rate**											
Low	1386 (20.9)	113 (1.7)	228 (3.4)	315 (4.8)	321 (4.9)	232 (3.5)	391 (5.9)	341 (5.2)	1295 (19.6)	1999 (30.2)	< 0.001
Average	1945 (30.2)	195 (3.0)	371 (5.8)	675 (10.5)	406 (6.3)	289 (4.5)	306 (4.7)	288 (4.5)	847 (13.1)	1127 (17.5)	
High	2331 (35.9)	241 (3.7)	431 (6.6)	991 (15.3)	433 (6.7)	248 (3.8)	267 (4.1)	273 (4.2)	611 (9.4)	668 (10.3)	
**Community illiteracy rate**											
Low	1164 (17.8)	100 (1.5)	220 (3.4)	258 (4.0)	286 (4.4)	215 (3.3)	373 (5.7)	374 (5.7)	1431 (21.9)	2105 (32.3)	
Average	2100 (32.2)	188 (2.9)	367 (5.6)	652 (10.0)	420 (6.4)	287 (4.4)	306 (4.7)	295 (4.5)	804 (12.3)	1112 (17.0)	< 0.001
High	2398 (36.9)	261 (4.0)	443 (6.8)	1071 (16.5)	454 (7.0)	267 (4.1)	285 (4.4)	233 (3.6)	518 (8.0)	577 (8.9)	
**Community unemployment**											
Low	1494 (22.3)	145 (2.2)	266 (4.0)	498 (7.5)	383 (5.7)	250 (3.7)	363 (5.4)	338 (5.1)	1239 (18.5)	1712 (25.6)	
Average	1834 (28.7)	197 (3.1)	363 (5.7)	748 (11.7)	401 (6.3)	260 (4.1)	279 (4.4)	285 (4.5)	822 (12.9)	1196 (18.7)	< 0.001
High	2334 (36.0)	207 (3.2)	401 (6.2)	735 (11.3)	376 (5.8)	259 (4.0)	322 (5.0)	279 (4.3)	692 (10.7)	886 (13.7)	
**Level 3 (State Level Characteristics)**											
**Rural proportion**											
Low rural proportion	574 (15.8)	28 (0.8)	63 (1.7)	67 (1.9)	134 (3.7)	110 (3.0)	171 (4.7)	202 (5.6)	881 (24.3)	1397 (38.5)	< 0.001
Average rural proportion	1337 (27.6)	125 (2.6)	221 (4.6)	409 (8.5)	263 (5.4)	199 (4.1)	259 (5.4)	229 (4.7)	747 (15.4)	1054 (21.8)	
High rural proportion	3751 (33.8)	396 (3.6)	746 (6.7)	1505 (13.6)	763 (6.9)	460 (4.2)	534 (4.8)	471 (4.3)	1125 (10.1)	1343 (12.1)	
**Health facility per 100,000**											
< 15	1494 (31.2)	146 (3.1)	274 (5.7)	692 (14.5)	363 (7.6)	184 (3.8)	199 (4.2)	190 (4.0)	544 (11.4)	704 (14.7)	< 0.001
15–25	3280 (30.0)	316 (2.9)	526 (4.8)	1022 (9.3)	568 (5.2)	412 (3.8)	520 (4.8)	501 (4.6)	1591 (14.5)	2203 (20.1)	
> 25	888 (23.2)	87 (2.3)	230 (6.0)	267 (7.0)	229 (6.0)	173 (4.5)	245 (6.4)	211 (5.5)	618 (16.1)	887 (23.1)	
**Distal Characteristic**											
**Region of residence**											
North Central	759 (23.8)	56 (1.8)	154 (4.8)	190 (5.9)	183 (5.7)	163 (5.1)	201 (6.3)	214 (6.7)	548 (17.2)	728 (22.8)	< 0.001
North East	1616 (38.0)	190 (4.5)	325 (7.7)	454 (10.7)	256 (6.0)	187 (4.4)	195 (4.6)	175 (4.1)	384 (9.0)	467 (11.0)	
North West	2239 (38.9)	216 (3.8)	403 (7.0)	1161 (20.2)	489 (8.5)	211 (3.7)	203 (3.5)	125 (2.2)	274 (4.8)	440 (7.6)	
South East	330 (17.0)	9 (0.5)	28 (1.4)	31 (1.6)	70 (3.6)	39 (2.0)	83 (4.3)	116 (6.0)	479 (24.7)	755 (38.9)	
South South	383 (17.4)	45 (2.0)	67 (3.0)	92 (4.2)	88 (4.0)	87 (3.9)	145 (6.6)	150 (6.8)	518 (23.5)	632 (28.6)	
South West	335 (15.2)	33 (1.5)	53 (2.4)	53 (2.4)	74 (3.4)	82 (3.7)	137 (6.2)	122 (5.5)	550 (24.9)	772 (34.9)	

**Fig. 3:**
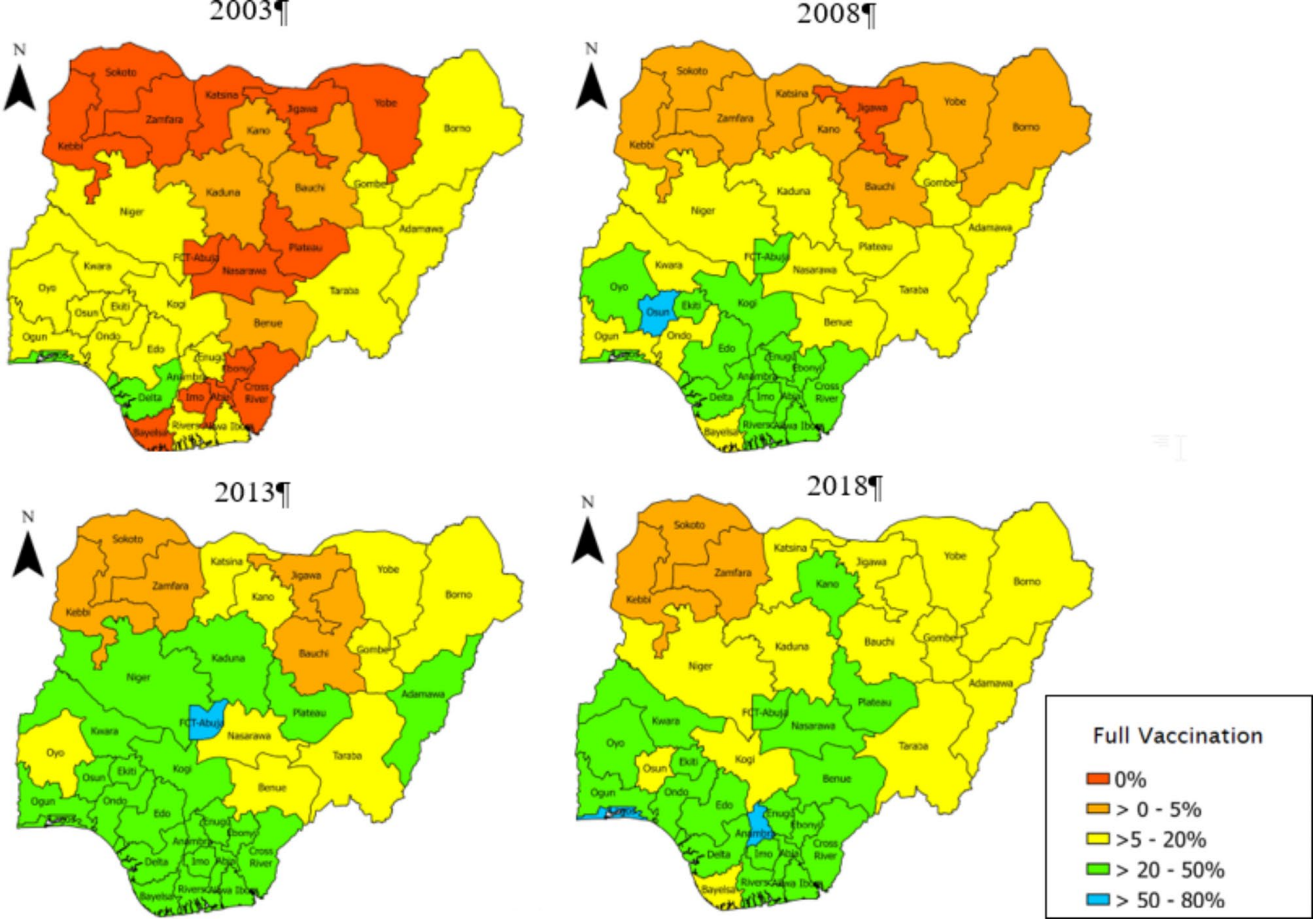
Spatial distribution of the prevalence of full vaccination across the survey years

Furthermore, Fig. 3 disaggregated the spatial prevalence of full vaccine uptake by each of the survey years. In 2003, a larger percentage of the states in the Northern region had a 0% prevalence of full vaccination, except Kano, Kaduna and Bauchi which had above 0% but less than 5% prevalence. Some states in the Southern region also had a 0% prevalence of full vaccination. The highest prevalence was observed in Lagos and Delta states.

In 2008, an improvement in full vaccine uptake was observed with only Jigawa reporting a 0% prevalence of full vaccination. Although there were improvements across states, only Osun had a prevalence above 50%. In 2013 and 2018, no state was found to have a 0% prevalence while Anambra and Lagos had the highest prevalence (> 50%) in 2013 and Abuja had above 50% full vaccine uptake in 2018. Overall Full vaccination coverage ranged from 1.7% in Sokoto to 51.9% in Anambra.

**Fig. 4:**
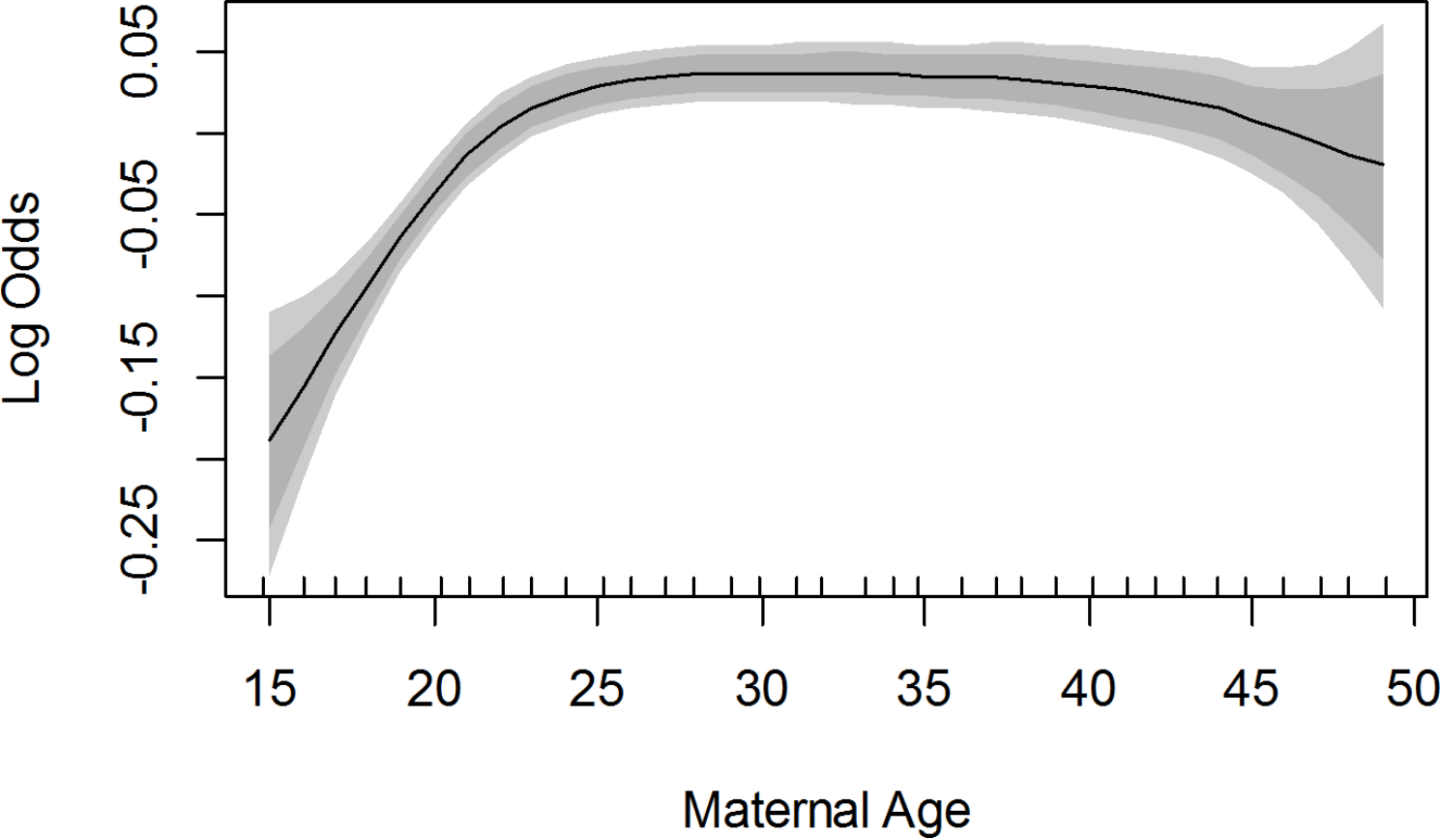
Posterior estimates of the smoothed posterior estimates showing the non-linear effects (log-odds) and 95% credible interval of mother’s age on the likelihood of vaccine uptake from the adjusted model for year and spatial effect

Figure 4 presents the smoothed map of the association between the age of mothers and vaccine uptake, adjusted for year and spatial effect. As the age of mothers increased, the likelihood of vaccine uptake also increased. Table 3 presents the output of smoothed Zero-inflated Poisson regression model for the number of doses of childhood vaccines taken among children aged 12–23 months in Nigeria, adjusted for spatial effects and other confounders.

**Table 3:** Zero-Inflated Poisson three-level variance components model showing the adjusted posterior likelihood of higher number of vaccine uptake

	POSTERIOR aIRR (95% CrI)
**CONTROL VARIABLE**	
**Year**	
2003	Reference
2008	1.08 (1.03–1.13)
2013	1.16 (1.11–1.21)
2018	1.22 (1.17–1.28)
**Level 1 (Individual Level Characteristics)**	
**Age**	
15–24 years	Reference
25–34 years	1.05 (1.03–1.07)
35–49 years	1.07 (1.05–1.10)
**Level of Education**	
No formal education	Reference
Primary education	1.11 (1.09–1.14)
Secondary/Tertiary education	1.16 (1.13–1.19)
**Religion**	
Christian	Reference
Islam	0.95 (0.93–0.97)
Others	0.83 (0.78–0.87)
**Working Status**	
Not currently working	Reference
Currently working	1.03 (1.01–1.04)
**Wanted child**	
Wanted then	Reference
Did not want then	0.99 (0.97–1.02)
**Attendant at delivery**	
Unskilled	Reference
Skilled	1.10 (1.09–1.12)
**Exposure to media**	
Not exposed at all	Reference
Partially exposed	1.01 (0.99–1.03)
Ver exposed	1.04 (1.02–1.07)
**Birth order of child**	
First	Reference
Second	0.99 (0.97–1.02)
Third	0.97 (0.95–0.99)
Fourth and above	0.97 (0.95–0.99)
**Wealth Status**	
Bottom 33%	Reference
Average	1.09 (1.07–1.12)
Top 33%	1.16 (1.13–1.19)
**Number of ANC visits**	
No visit	Reference
1–3 visits	1.16 (1.13–1.19)
4–7 visits	1.23 (1.21–1.26)
8 or more visits	1.20 (1.18–1.23)
**Sex of the child**	
Male	Reference
Female	1.00 (0.99–1.01)
**Healthcare decision maker**	
Self	
Husband alone	0.99 (0.97–1.01)
Joint	1.01 (0.98–1.03)
Other	0.98 (0.95–1.02)
**Level 2 (Community Level Characteristics)**	
**Place of residence**	
Urban	Reference
Rural	0.99 (0.97–1.01)
**Community poverty rate**	
Low	Reference
Average	1.04 (1.02–1.06)
High	1.01 (0.99–1.04)
**Community illiteracy rate**	
Low	Reference
Average	0.99 (0.97–1.02)
High	0.96 (0.94–0.99)
**Community unemployment**	
Low	Reference
Average	1.03 (1.01–1.04)
High	0.96 (0.93–0.99)
**Level 3 (State Level Characteristics)**	
**Rural proportion**	
Low rural proportion	Reference
Average rural proportion	0.94 (0.86–1.03)
High rural proportion	0.91 (0.83–1.01)
**Health facility per 100,000**	
< 15	Reference
15–25	1.02 (0.93–1.13)
> 25	1.04 (0.93–1.16)
**RANDOM EFFECTS**	
**Individual-level Variance**	
Mean (95% CrI)	0.0058 (0.0009)
**Community-level Variance**	
Mean (95% CrI)	0.0020 (0.0016)
**State-level Variance**	
Mean (95% CrI)	0.0021 (0.0018)

There was a significant variation in the number of doses of childhood vaccines taken, across communities, and also across states. The community-level variance was estimated as 0.0020, and 0.0021 at the state level. The credible intervals of these estimates are significant, so the hypothesis that the regression slopes for the number of doses of childhood vaccines taken vary across the individual-, community- and state-level is supported by the data although the majority of variations can be attributed or explained by individual-level effects (0.0058).

In 2008, 2013 and 2018 survey years, after adjusting for spatial effects and other confounders, children had a higher likelihood of a higher number of doses of childhood vaccines taken than in 2003. Children whose mothers were aged 25–34 years and 35–49 years age group had an 8% higher likelihood of a higher number of doses of childhood vaccines taken relative to those aged 15–24 years (adjusted Incidence “risk” Ratio (aIRR: 1.05, 1.07; 95% credible interval (CrI): 1.03–1.07, 1.05–1.10). Similarly, an increase in the level of formal education increased the incidence of vaccine uptake; children whose mothers had primary education or secondary/tertiary education had a higher incidence than those whose mothers had no formal education (aIRR: 1.11, 1.16; 95% CrI: 1.09–1.14, 1.13–1.19).

Individuals from better-off households had a higher number of doses of childhood vaccines taken than those individuals from households in poorer categories (aIRR: 1.09, 1.16; 95% CrI: 1.07–1.12, 1.13–1.19 respectively), and those who had between 1 and 3 antenatal care (ANC) visits, 4–7 ANC visits and 8 or more ANC visits had a higher incidence of vaccine uptake than those who never attended ANC services (aIRR = 1.16, 1.23, 1.20; 95% CrI = 1.13–1.19, 1.21–1.26 and 1.18–1.23 respectively). Other significant factors associated with the number of doses of childhood vaccines taken are working status, religion, attendant on delivery (which acted as a surrogate for postnatal care attendance), religion and birth order.

Children whose mothers lived in communities with average poverty rates have a higher likelihood of vaccinating their children (aIRR: 1.04; 95% CrI: 1.02–1.06) and communities with high illiteracy rates had lower odds of child vaccine uptake (aIRR: 0.96; 95% CRI: 0.94–0.99). The high unemployment rate in communities corresponded to a lower likelihood of vaccine uptake (aIRR: 0.96; 95% CrI: 0.93–0.99). Communities with average unemployment rates had a higher likelihood of child vaccine uptake than communities with low unemployment (aIRR: 1.03; 95% CrI: 1.01–1.04) while communities with high unemployment rates had a lower likelihood (aIRR: 0.96; 95% CrI: 0.93–0.99).

**Fig. 5:**
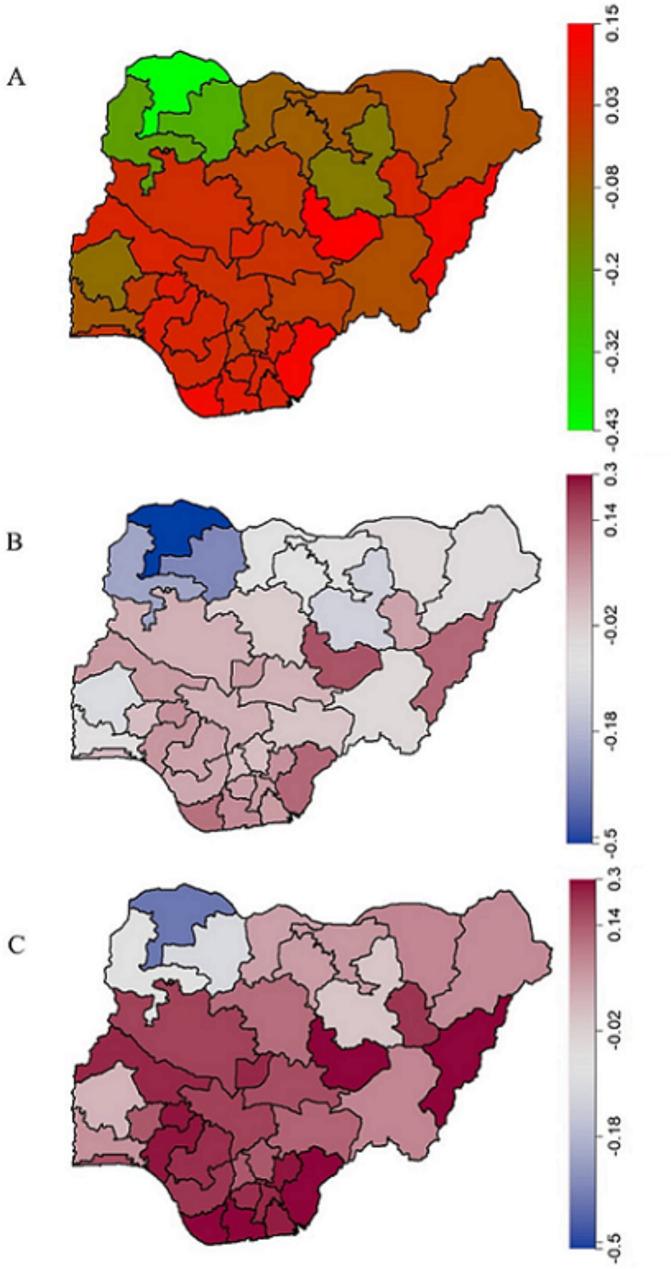
Adjusted Total Spatial Effects (**A**) with the 2.5% (**B**) and 97.5% (**C**) posterior estimates significance map for vaccine uptake among children aged 12–23 months in Nigeria

Notably, at the state level, none of the variables considered was significantly associated with the number of doses of childhood vaccines taken. However, the posterior median map in Fig. 5 (adjusted for spatial effects) showed low uptake of vaccines in the Northern region compared to a high likelihood in the Southern region.

## Discussion

There was a general improvement in vaccine uptake over time, as the percentage of children who were fully vaccinated increased between 2003 and 2018 just as the average number of doses of childhood vaccines received increased during the same period. This was in agreement with a previous study [34], but at variance with findings from other African countries such as Burundi, Rwanda and Kenya [11, 35, 36]. Despite the observed increase in vaccine uptake, the percentage of non-vaccinated children remained high. A persistently low uptake was observed in the Northern region of the country throughout the period covered in this study (2003–2018).

Maternal age was found to be a very important socio-economic and individual-level predictor of the number of doses of childhood vaccines taken, with higher maternal age associated with an increased number of childhood vaccine doses taken. As mothers increased in age, they plausibly would have had prior childbirth experience and known the importance of vaccines. This stance was also supported by previous studies [11, 37].

Also, the mother’s educational level was positively correlated with the number of doses of childhood vaccines taken; a higher level of education corresponds to an increased number of doses of childhood vaccines taken. Mothers who were educated would be well informed of the relevant benefits and advantages of taking vaccines, which in turn leads to an increase in uptake. Similar results were found in previous studies, as educated mothers (which may be extended to occupation) are more informed on issues relating to family planning and child health [10, 38]. Household wealth status was found to significantly predict vaccine uptake as women from households in the richer quantiles may be more educated and have well-paying jobs than in poorer households [10]. This could be ascribed to higher awareness and education among women in richer households than the poorer ones. Further recent studies carried out in LMICs have identified an association between the financial and educational level of women and their uptake of vaccines for their children [39–42].

Religion is a very important determinant of health-seeking behaviours and health outcomes [10], as evidenced by the study. It is not unlikely that some religion promote vaccination more than others. Bearing in mind that the North, with the lowest vaccine uptake, is predominantly Muslims and of Hausa/Fulani culture, poor vaccine uptake in that zone could be ascribed to cultural hesitance and religious influence. In 2022, with evidence from 66 LMICs, Santos et al. established that religious affiliation is a significant driver of childhood vaccine uptakes.

Having a skilled birth attendant increased the likelihood of a child receiving all immunizations at birth; this is also reflected in an increased incidence of vaccine uptake among women who attended antenatal care, plausibly the trust in the health system is increased by previous experience. Attendance of antenatal and postnatal clinics has been linked with increased confidence and a positive inclination towards the health system [12].

The association between the number of doses of childhood vaccines taken and access to media was significant. Several studies have suggested that regular exposure through mass media and other community dissemination platforms are key channels for promoting vaccines [14, 38, 43]. Birth order was significantly associated with vaccine uptake and findings from previous literature have reported that increased demand in competition for family resources is associated with an increased number of children [44]. The relationship between these maternal characteristics and the number of doses of childhood vaccines taken indicates the importance of media access and family planning in the uptake of childhood vaccines.

Previous studies have linked health facility access and place of residence (rural and urban) to health outcomes, including vaccine uptake [10, 14, 45, 46]. Although the place of residence was not significant in the current study, other variables such as community poverty, illiteracy and unemployment are a direct derivation of residence are associated with specific place of residence. We can infer then, that residence in the rural areas can impact vaccine uptake. Notably, the gender of the children did not affect the uptake of vaccines, as other studies pointed out in Nigeria and Ghana, but not in some other countries like India [10, 11, 47].

Children whose mothers reside in communities with high rural illiteracy and unemployment rates are at a lower likelihood of a high number of doses of childhood vaccines taken. As supported by Cata-Preta, this can be attributed to poor awareness and lower access to health services in rural areas (possibly by distance), and an insufficient number of facilities to cover the large population in those areas [48]. Although state-level characteristics were not significant in this study, we postulate that children whose mothers reside in states with a high rural proportion had the likelihood of taking a lower number of doses of childhood vaccines. This can be attributed to lower access to health services in rural areas from community-level variables – plausibly by distance and an insufficient number of facilities to cover the disadvantaged areas.

## Strength and limitations

A major strength of this study was that the study pooled set of representative data between 2003 and 2018 in Nigeria to model the number of doses of childhood vaccines taken among children aged 12–23 months in Nigeria. Furthermore, this study applied the Bayesian framework against the frequentist approach in establishing the number of doses of childhood vaccines taken. Also, we assessed the hierarchical nature of the data as well as the spatial-temporal distribution of the vaccine uptakes. However, a major drawback of this study is the cross-sectional design which is limited in establishing causality. Also, there may have been under-reporting in the number of doses of childhood vaccines taken; the NDHS assumes that vaccination has not been obtained when the record of vaccination was missing or the mother does not remember if the child has taken the vaccine. The secondary nature of the data also limited the choices of explanatory variables.

## Conclusion

Different individual-, community-, and state-level variations were observed to significantly affect vaccine uptake among children aged 12–23 months in Nigeria. Improvements in vaccine uptake were recorded across the survey years and the analysis showed that vaccine uptake differed across the individual-level (including age, education, religion, occupation, attendant on delivery, exposure to media, wealth, antenatal visits) and communities (poverty, illiteracy and unemployment rates). This points to the influence of demographic, socio-economic and environmental factors, as well as cultural factors. The posterior prediction, from evidence in the estimates, poses a stern call to action to prevent a reduction in the number of future vaccine uptake.

Furthermore, since vaccines have been proven to be an effective strategy in reducing child morbidity and mortality across countries of the world, programmes aimed at educating women on the benefits of hospital delivery and vaccines should be implemented to increase uptake. There is a need to improve overall community awareness of vaccination and special intervention will be neccessary where the numbers of vaccines received are low. The northern states may benchmark what practices enhance higher uptake in the south. Government should involve non-governmental organizations and other relevant organizations in promoting and sustaining vaccine uptake among children, especially in locations where uptake is low.

### Electronic supplementary material

Below is the link to the electronic supplementary material.

Supplementary Material 1

## Data Availability

Data used for this study are available on the DHS website, https://dhsprogram.com.

## List of abbreviations

LMIC: Low- and middle-income countries
NPHCDA: National Primary Health Care Development Agency
NDHS: National Demographic and Health Survey
BCG: Bacillus-Calmette-Guérin
DPT: Diphtheria-Pertussis-Tetanus
GLMM: Generalized linear mixed model
CrI: Credible Interval
IRR: Incidence risk ratio
ANC: Antenatal care
