# Supplementary material for: Spatio-temporal analysis of childhood vaccine uptake in Nigeria: a hierarchical Bayesian Zero-inflated Poisson approach
Source: BMC Pediatr. 2023 Sep 29;23:493. doi: 10.1186/s12887-023-04300-x (PMC10540393; doi:10.1186/s12887-023-04300-x)
Supplement: Supplementary file 1 — Supplementary Material 1 [file 12887_2023_4300_MOESM1_ESM.docx]

Title: Model Diagnostics and Selection

|  | **-2 Log Likelihood** | **LOOIC** | **WAIC** |
| --- | --- | --- | --- |
| **Poisson** | -31384.02 | 63586.2 | 64503.7 |
| **Zero Inflated Poisson** | -27235.46 | 54601.6 | 54735.4 |
